# SG-APSIC1155: Drug susceptibility patterns of fulminant group G *Streptococcus* (GGS) infection as a re-emerging infectious disease in Japan

**DOI:** 10.1017/ash.2023.32

**Published:** 2023-03-16

**Authors:** Masaaki Minami, Ryoko Sakakibara, Shunsuke Akahori

**Affiliations:** Nagoya City University, Japan; Nagoya, Daido Hospital, Japan; Nagoya, Nagoya City University, Japan

## Abstract

**Objectives:** Severe streptococcal infections are invasive, re-emerging infections that rapidly worsen and lead to death. Not only group A *Streptococcus* (GAS) but also group g *Streptococcus* (GGS) are the causative agents of this infection. Moreover, GGS produces hemolytic toxins, proteolytic toxins, and other toxins like GAS. Furthermore, drug-resistant *Streptococcus* spp, like other pathogenic bacteria, are on the rise worldwide. However, drug resistance has not been studied extensively in invasive GGS. Therefore, we investigated the drug susceptibility of GGS clinical isolates that are closely related to fulminant streptococcal infections. **Methods:** We used GGS strains isolated from sterile sites of invasive infections at a hospital in Nagoya City, Japan, from 2017 to 2021. Bacterial identification and drug-susceptibility testing were performed using a VITEK-2 system. **Results:** Overall, 53 strains were included in the study. The GGS strains examined in this study were resistant to 3 different antibiotics (erythromycin, clindamycin, and minocycline). Also, 18 strains (34%) were resistant to erythromycin, 9 (17%) were resistant to clindamycin, and 18 (34%) were resistant to minocycline. Moreover, there were 5 strains (9.4%) of 2-drug–resistant bacteria and 8 strains (15.1%) of 3-drug–resistant bacteria. **Conclusions:** Acquired resistance not only to individual antibiotics but also to multiple antibiotics suggests that GGS tends to become multidrug resistant. Continued surveillance of the drug susceptibility of GGS as a potential cause of fulminant streptococcal infections will be necessary in the future.

